# Microdistribution of Faunal Assemblages at Deep-Sea Hydrothermal Vents in the Southern Ocean

**DOI:** 10.1371/journal.pone.0048348

**Published:** 2012-10-29

**Authors:** Leigh Marsh, Jonathan T. Copley, Veerle A. I. Huvenne, Katrin Linse, William D. K. Reid, Alex D. Rogers, Christopher J. Sweeting, Paul A. Tyler

**Affiliations:** 1 Ocean and Earth Science, National Oceanography Centre, University of Southampton, Southampton, United Kingdom; 2 Marine Geoscience, Natural Environment Research Council, National Oceanography Centre Southampton, Southampton, United Kingdom; 3 British Antarctic Survey, High Cross, Cambridge, United Kingdom; 4 School of Marine Science & Technology, Newcastle University, Newcastle upon Tyne, United Kingdom; 5 Department of Zoology, University of Oxford, Oxford, United Kingdom; Institute of Marine Research, Norway

## Abstract

Chemosynthetic primary production by microbes supports abundant faunal assemblages at deep-sea hydrothermal vents, with zonation of invertebrate species typically occurring along physico-chemical gradients. Recently discovered vent fields on the East Scotia Ridge (ESR) in the Southern Ocean represent a new province of vent biogeography, but the spatial dynamics of their distinct fauna have yet to be elucidated. This study determines patterns of faunal zonation, species associations, and relationships between faunal microdistribution and hydrothermal activity in a vent field at a depth of 2,400 m on the ESR. Remotely operated vehicle (ROV) dives obtained high-definition imagery of three chimney structures with varying levels of hydrothermal activity, and a mosaic image of >250 m^2^ of seafloor co-registered with temperature measurements. Analysis of faunal microdistribution within the mosaiced seafloor reveals a consistent pattern of faunal zonation with increasing distance from vent sources and peak temperatures. Assemblages closest to vent sources are visibly dominated by a new species of anomuran crab, *Kiwa* n. sp. (abundance >700 individuals m^−2^), followed by a peltospiroid gastropod (>1,500 individuals m^−2^), eolepadid barnacle (>1,500 individuals m^−2^), and carnivorous actinostolid anemone (>30 individuals m^−2^). Peripheral fauna are not dominated by a single taxon, but include predatory and scavenger taxa such as stichasterid seastars, pycnogonids and octopus. Variation in faunal microdistribution on chimneys with differing levels of activity suggests a possible successional sequence for vent fauna in this new biogeographic province. An increase in δ^34^S values of primary consumers with distance from vent sources, and variation in their δ^13^C values also indicate possible zonation of nutritional modes of the vent fauna. By using ROV videography to obtain a high-resolution representation of a vent environment over a greater extent than previous studies, these results provide a baseline for determining temporal change and investigations of processes structuring faunal assemblages at Southern Ocean vents.

## Introduction

The abundant faunal assemblages colonising deep-sea hydrothermal vents are fuelled predominantly by chemosynthetic microbial primary production, typically using the oxidation of reduced inorganic compounds such as hydrogen sulfide at the mixing interface between hydrothermal vent fluids and seawater [1]. Vent fauna utilise microbial primary production by direct consumption of free-living microorganisms (either filter feeding or grazing), through symbiotic relationships with microorganisms (either endosymbiotic or ectosymbiotic), or through a combination of both nutritional modes [2]. Consequently, primary consumers often occur with extremely high population densities compared with non-chemosynthetic deep-sea environments, generally covering all available surfaces around vent fluid exits [3], [4]. A main objective in ecological studies is to understand the factors determining the distribution and abundance of individual populations [5], and in dynamic environments such as hydrothermal vents, determining faunal distributions is a starting point for developing ecological understanding [6], [7], [8]. Quantifying the composition and microdistribution of faunal assemblages at vents is also a prerequisite for understanding temporal patterns such as succession (e.g., [9], [10], [11], [12]), and the impact of anthropogenic activities such as the mining of seabed massive sulfide (SMS) deposits [13].

Within a single hydrothermal vent field, distribution patterns of species often match gradients in physicochemical conditions over spatial scales from a few centimetres to tens of metres or greater [9], [14]. Zonation of faunal assemblages at deep-sea hydrothermal vents therefore occurs over similar spatial scales to those found on rocky intertidal shores [15]. Proximity to and tolerance of vent effluent may be a primary factor in determining the extent to which species can exploit chemosynthetic primary production. Consequently, physicochemical factors appear to play important roles in determining the distribution patterns of species at vents [11], [16], [17], [18], [19], [20]. The development of vent ecology has therefore mirrored that of rocky intertidal ecology [21], initially considering physical tolerance of conditions as a primary determinant of zonation patterns, and subsequently investigating the role of biological interactions (e.g., [11], [22], [23], [24]). Unlike the rocky intertidal environment, however, acquiring primary observational data at deep-sea vents is hampered by the relative inaccessibility of the environment, and refinements in the use of deep-submergence platforms are required to overcome this limitation [25], [26].

**Figure 1:**
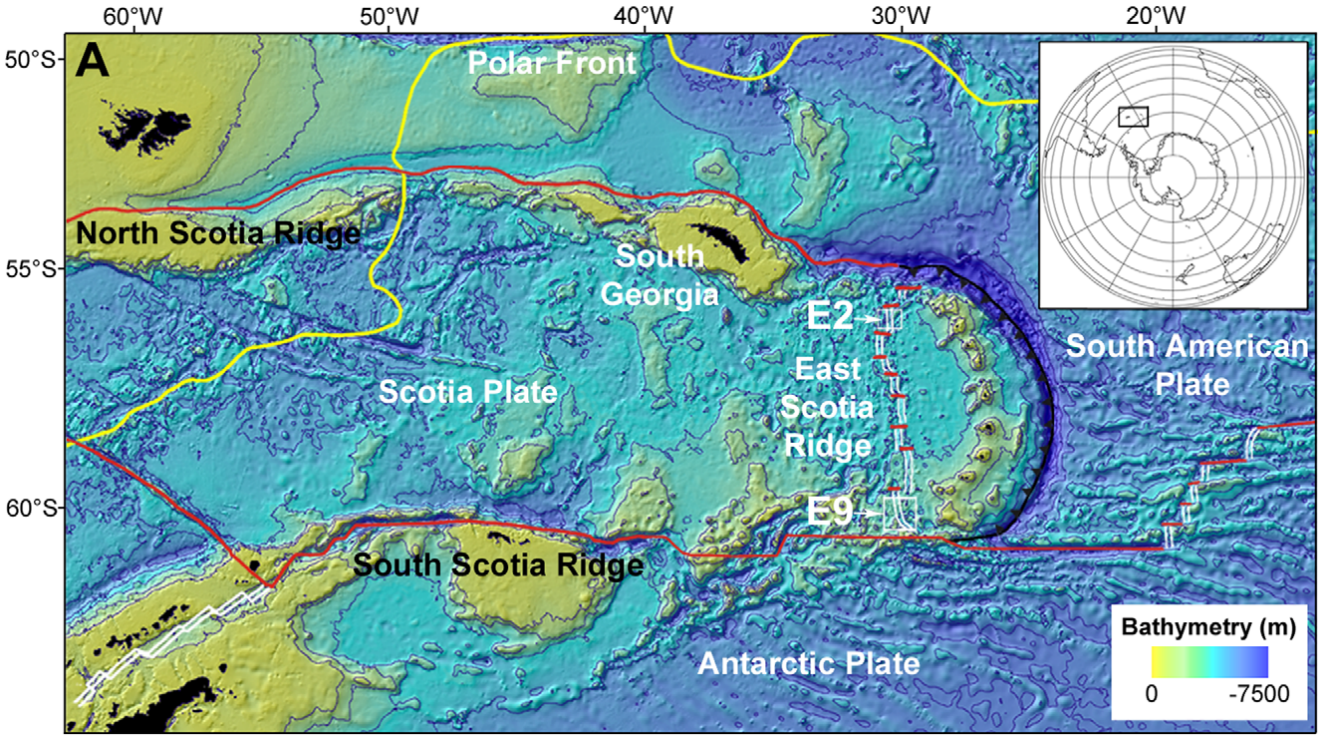
Location of the E2 and E9 hydrothermal vent fields on the East Scotia Ridge (ESR) back-arc basin, Southern Ocean.

**Figure 2:**
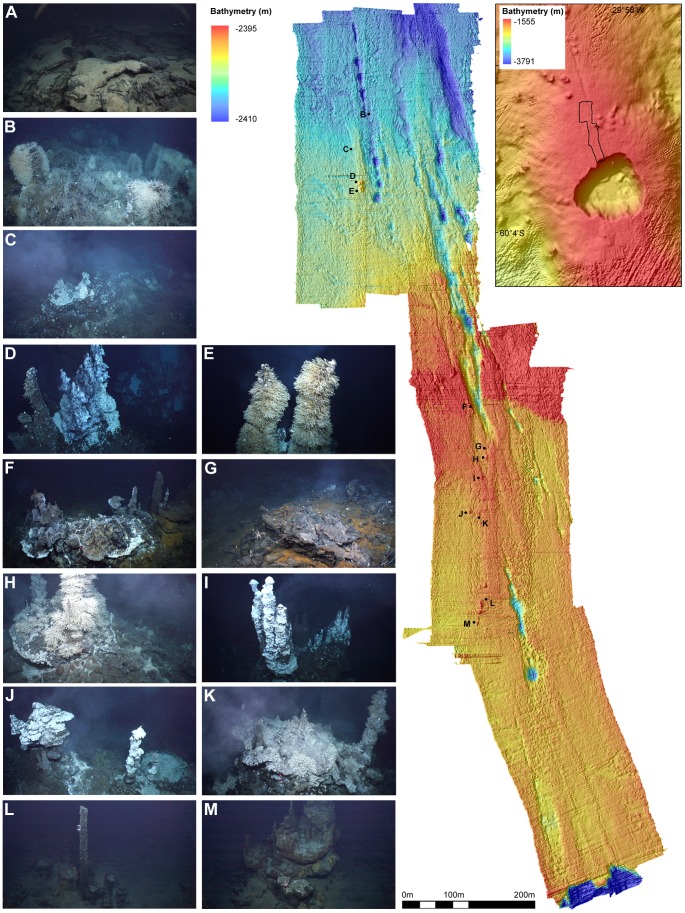
The E9 hydrothermal vent field. (Top-right) Ship acquired swath bathymetry of the ‘Devils Punchbowl’ collapsed caldera. Black outline denotes the E9 vent field and area of ROV-based swath. (Main bathymetric map) High-resolution ROV-acquired mulitbeam bathymetry of the E9 vent field. Waypoints denote areas of interest (A) Flat sheet lavas, typical background substrate of the E9 vent field (B) Most northern point of low-lying hydrothermal activity (C) “Twin Peaks” (D) “Black and White” (E) “Carwash” (F) “Temple” (G) “Marshland” (H) “Marsh Towers”; (I) “Ivory Towers” chimney complex (J) “Pagoda” chimney complex (K) “Launch Pad” chimney complex (L) “Needle” (M) “Windsor Castle”.

Since the first direct observations of deep-sea hydrothermal vent fields in the late 1970 s, several biogeographic provinces have been defined for vent fauna, which differ in the species composition of their assemblages [27], [28], [29], [30]. The composition and microdistribution of assemblages have been studied at vent fields in these established provinces, but with differences in effort that reflect the history of vent exploration. In the eastern Pacific Ocean, there have been numerous studies of faunal microdistribution at vent fields on the East Pacific Rise and Juan de Fuca Ridge [9], [11], [15], [16], [17], [18], [31], [32], [33], [34], [35], [36], [37], [38], [39], [40], [41], [42], [43], [44], but fewer studies so far on the Mid-Atlantic Ridge [12], [19], [45], [46], [47], [48], [49] and in Western Pacific back-arc basins [50], [51].

**Figure 3:**
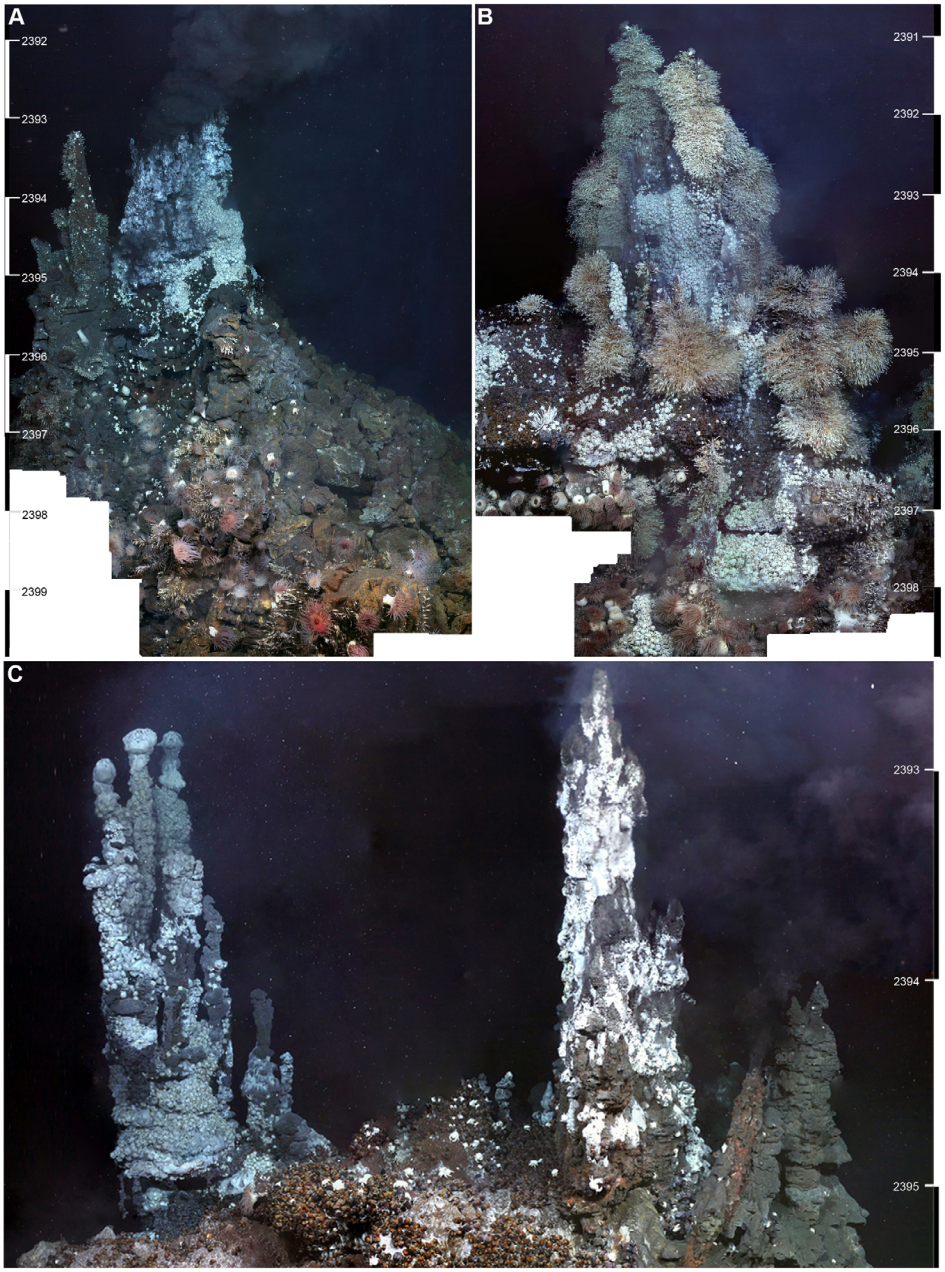
Vertical mosaic images created using sequential image captures from the 1080i video footage (A) “Black & White” 9.7m edifice (−60°02.568, −29°58.905). Dive 140, 2400 m depth, ROV heading 090° (B) “Carwash” 9.7 m edifice (−60°02.572, −29°58.904). Dive 140, 2401 m depth, ROV heading 128° (C) “Ivory Towers” 6.7 m edifice (−60°02.809, 29°58.708). Dive 142, 2395 m depth, ROV heading 090°.

Exploration of the East Scotia Ridge (ESR), an intermediate-rate back-arc spreading centre in the Southern Ocean [52] (Figure 1), has recently revealed vent fields inhabited by new, undescribed species of anomuran crab, eolepadid barnacle, lepetodrilid and peltospiroid gastropods, actinostolid anemones, and a stichasterid seastar, which represent a new province of vent biogeography [53]. Here we: (i) describe the distribution of vent structures in the newly-discovered E9 vent field on the ESR; (ii) determine patterns of faunal zonation and species association in the E9 vent field, using refinements in videography from a deep-sea remotely operated vehicle (ROV); and (iii) examine relationships between faunal microdistribution, vent activity, and the trophic ecology of abundant primary consumers using carbon and sulphur stable isotope analyses. These studies therefore provide a first characterisation of faunal ecology in this new province of vent biogeography.

**Figure 4:**
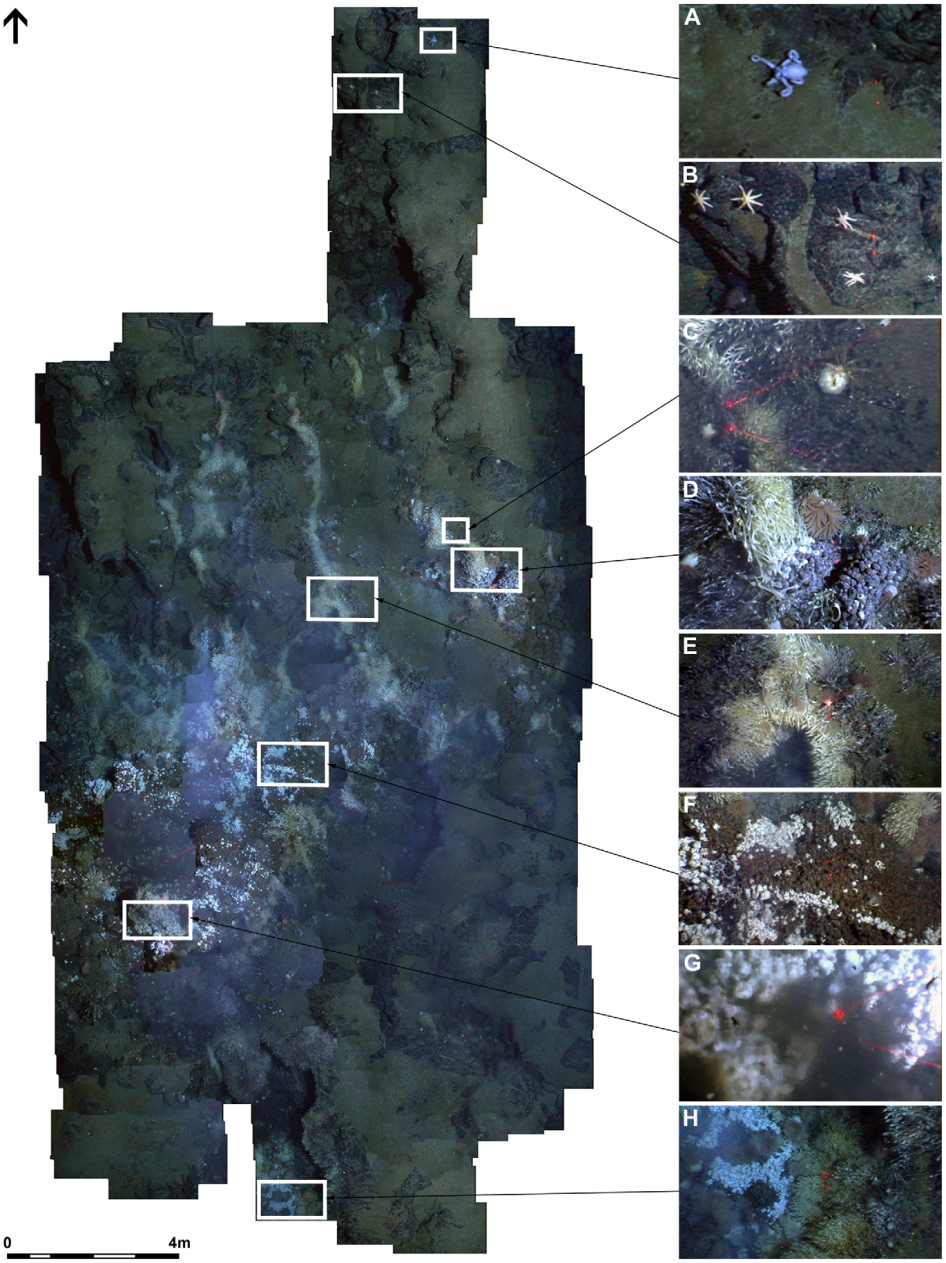
Horizontal mosaic of an area of low-lying diffuse flow around “Twin Peaks”. Dive 139, 2401 m depth. ROV heading 358°. Area mosaiced ∼280 m^2^. Laser scale = 10 cm (A) unidentified Octopod (B) cluster of stichasterid seastars (C) pycnogonid genus *Colessendeis* sp. predating on actinostolid (D) small area of diffuse flow (E) barnacles on ‘y’ – shaped fracture (F) “*Kiwa assemblage B*” and “*gastropod assemblage*” (G) “*Kiwa assemblage A*” associated with peak ROV mounted CTD temperature measurement (6.03°C) (H) small area of diffuse flow in collapsed basalt.

## Materials and Methods

### Image Acquisition and Mosaicing

During research cruise 42 of the *RRS James Cook* (7^th^ January –24^th^ February 2010) the *Isis* ROV completed 9 dives with a total bottom time of 96 hours at the E9 vent field on the ESR (Figure 2). These dives included systematic videographic surveys of selected areas of the vent field. Surveys of horizontal substratum were undertaken using a downward-looking (seabed-perpendicular) 3-chip CCD (charge-coupled device) video camera (Insite Pacific Atlas). Two lasers, 0.1 m apart, were mounted parallel to the focal axis of the camera to provide scale in images. A CTD mounted to the starboard side of the ROV recorded water temperature at an altitude of 2 to 3 m above the seafloor during these horizontal mosaic surveys.

**Figure 5:**
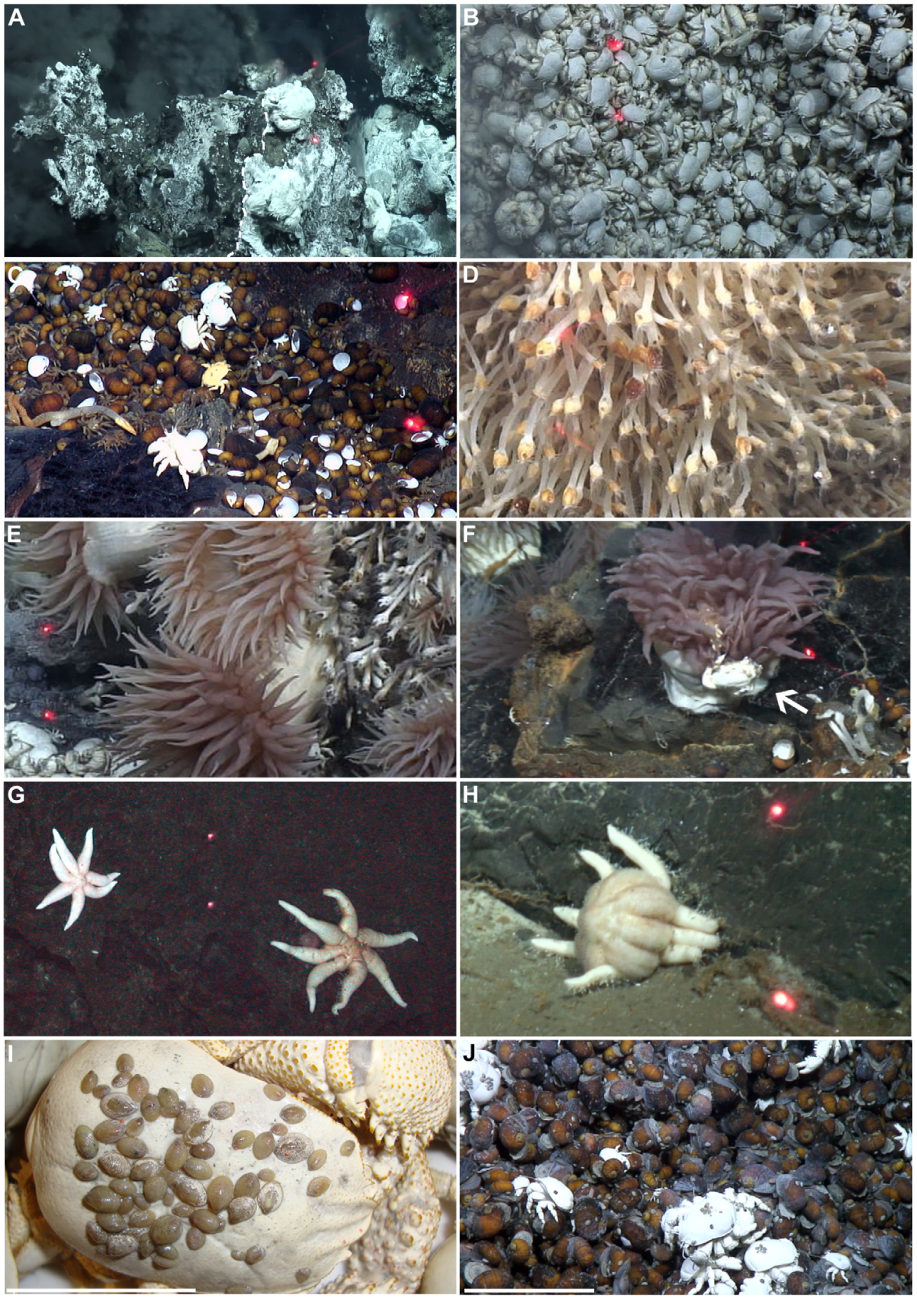
Images captured from high-definition video footage of the key fauna from the E9 vent field. Unless stated otherwise, laser scale or scale bar = 10 cm (A) *Anhydrite assemblage* adjacent to *Kiwa assemblage A* (B) *Kiwa assemblage B* (C) *gastropod assemblage* (D) *barnacle assemblage* (E) *anemone assemblage* (F) Actinostolid observed egesting a *Kiwa* n. sp. carapace (indicated by white arrow) (G) Un-described seven-armed stichasterid seastar (H) Predatory seastar observed preying on a *Kiwa* n. sp. (I) On-board still image of *Lepetodrilus* n. sp. on carapace of large individual of *Kiwa* n. sp. 62 individuals counted, Scale = 5 cm (J) *Lepetodrilus* n. sp in association with the gastropod assemblage (5900–6200 individuals m^−2^).

**Table 1:** Faunal composition of the seven identified assemblages from the E9 vent field, Southern Ocean.

					Kiwa Assemblage							
				Anhydrite Assemblage	A	B	C	Gastropod Assemblage	Barnacle Assemblage	Anemone Assemblage	Peripheral Assemblage	Substratum Assemblage
**Cnidaria**	Anthozoa	*Pacmanactis* spp	white solid base, pink tentacles	–	–	–	–	–	(+)	++	–	(+)
		cf *Marianactis* sp.	white small	–	–	–	–	–	(+)	+	–	(+)
**Mollusca**	Gastropoda	Peltospiroidea n sp.		–	–	–	–	++	–	–	–	(+)
		*Lepetodrilus* sp.		–	+	+	(+)	+	++	(+)	–	–
		Octopodidiae		–	–	–	–	–	–	–	(+)	–
**Arthropoda**	Cirripedia	*Vulcanolepas* n. sp.	(larger size)	–	–	–	–	–	++	(+)	–	(+)
		*Vulcanolepas* n. sp.	(juv. seedings)	–	–	–	–	–	–	–	–	(+)
	Anomura	*Kiwa* n.sp.	(carapace47±0.8 mm)	(+)	++	(+)	–	–	–	–	–	–
		*Kiwa* n.sp.	(carapace30±0.8 mm)	-	(+)	++	(+)	(+)	(+)	–	(+)	(+)
		*Kiwa* n.sp.	(carapace12±0.4 mm)	-	-	(+)	++	-	(+)	–	-	(+)
	Pycnogonida	*Sericosura* spp		–	–	–	–	+	–	–	–	–
		*Colossendeis* spp		–	–	–	–	–	–	–	(+)	–
**Echinodermata**	Asteroidea	Seven-armed Stichasteridae		–	–	–	–	–	–	–	(+)	–
		*Freyella* cf. *fragilissima*		–	–	–	–	–	–	–	(+)	–
**Chordata**	Vertebrata	Zoarcid fish		–	–	–	–	–	–	–	(+)	–
**Micro-organisms**		Visible microbial mats		(+)	++	+	–	–	–	–	–	(+)
**Flow Features**		Proximity of Black Smoker		++	++	+	–	+	–	–	–	–
		Proximity of flange/diffusion zone		++	++	+	(+)	+	+	+	–	–
		In visible diffuse flow		++	++	++	(+)	++	+	+	–	–
**Geochemical**		Anhydrite		++	++	(+)	(+)	–	–	–	–	–

Physico-chemical parameters and locality of assemblages are also identified for a black smoker assemblage (based on “Black & White”). Mega- and macro- fauna were identified using high-definition video and mosaic imagery, and were taxonomically verified from ROV-collected voucher specimens. ++, Abundant; +, present; (+) occasionally present; – absent.

Surveys of upstanding structures such as vent chimneys were undertaken using a 1080i high-definition video camera (Insite Pacific Mini-Zeus) on a pan-and-tilt mount. For these surveys, this camera was configured to view horizontally forwards from the ROV, so that its focal axis was perpendicular to the vertical plane described by the movement of the vehicle during surveys. Once configured, the pan and tilt module was then fixed for the duration of the survey. Two lasers, 0.1 m apart, were mounted parallel to the focal axis of the camera to provide scale in images. Continuous CTD measurements of water temperature during imaging of vent chimneys cannot be related to the surfaces surveyed, because of the vertical advection of vent fluids.

**Figure 6:**
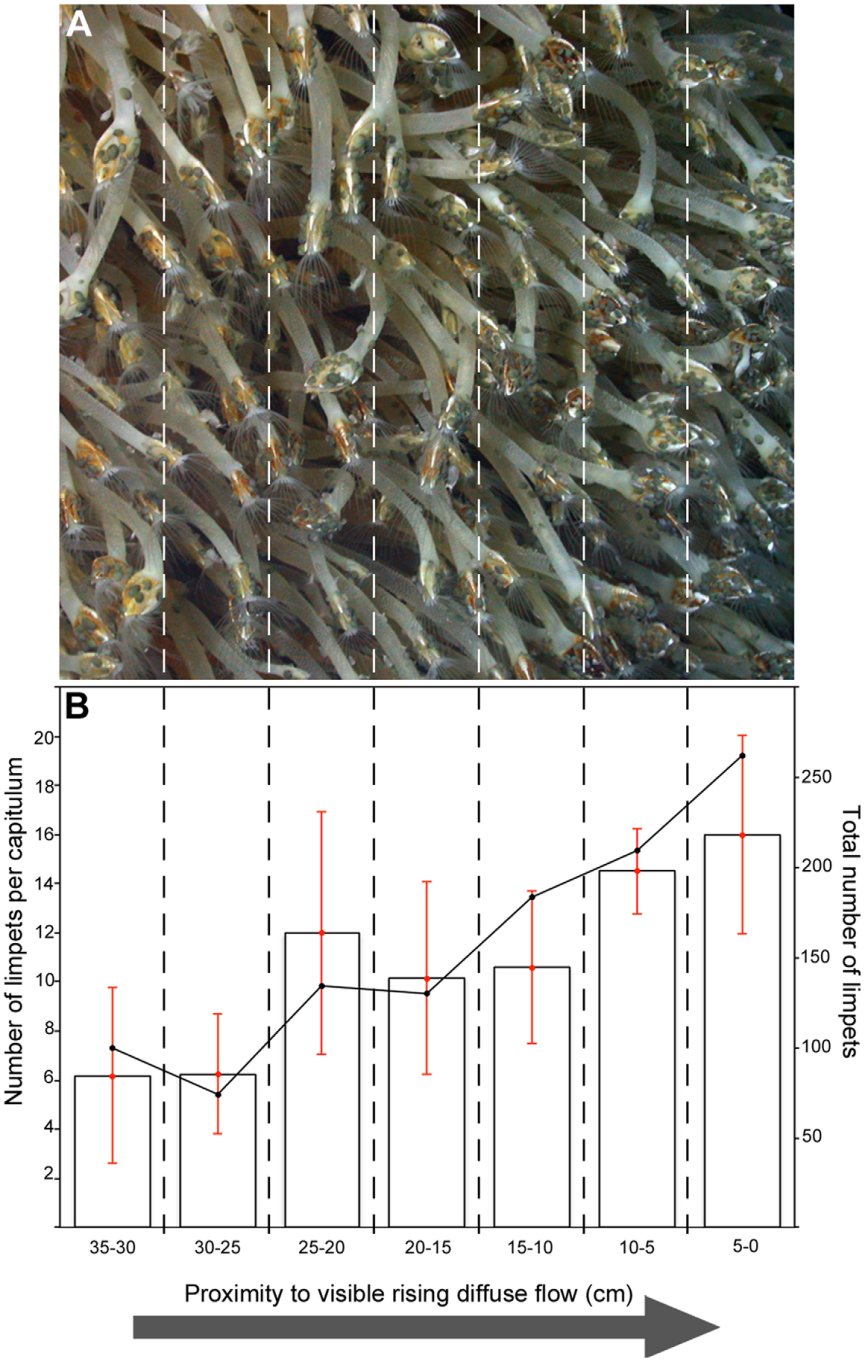
*Lepetodrilus* n. sp. (A) Barnacle assemblage with associated *Lepetodrilus* n. sp. 20,172–56,904 individuals m^−2^. (B) Line plot indicates the total number of limpets in each 5 cm increment towards an area of rising hydrothermal effluent. Bar plot indicates average number of limpets per capitula. Error bars are standard deviation.

Several criteria constrained the selection of edifices for vertical mosaicing surveys. As a result of ROV dive time limitations, only three upstanding structures could be surveyed. These three chimneys were selected to represent a range of levels of hydrothermal activity. For inter-chimney comparisons, the west face (approximate ROV heading 090°) was chosen, as this heading was most amenable to complete ROV transect lines in a vertical plane with no obstructions. Selecting the same cardinal face also minimises possible variations between faces that may result from the effects of background currents. These criteria resulted in the following three structures being surveyed: “Black & White”, “Carwash” and “Ivory Towers” (Figure 3).

**Figure 7:**
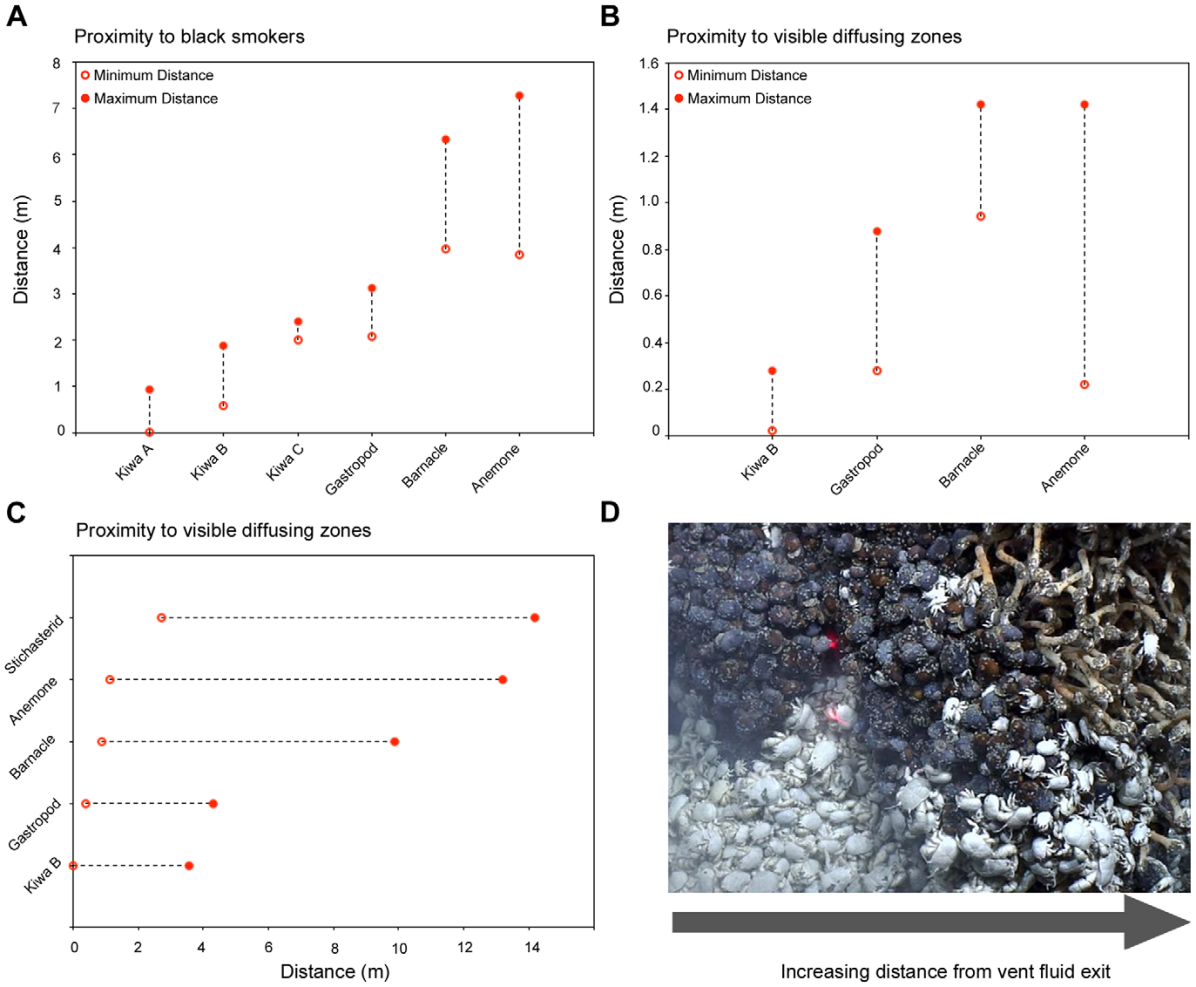
Minimum and maximum distance of assemblages from varying fluid exits. (A) Assemblage distances from the vertical high-temperature black smoker exit on the “Black & White” edifice (B) Assemblage distances from the vertical diffuse flow exits on the “Carwash” edifice. (C) Assemblage distances from the horizontal diffuse flow exit indicated by ROV CTD peak temperature measurement on the “Twin Peaks” mosaic (see Figure 4 & Figure 8). (D) Image illustrating transition from assemblage to assemblage.

**Figure 8:**
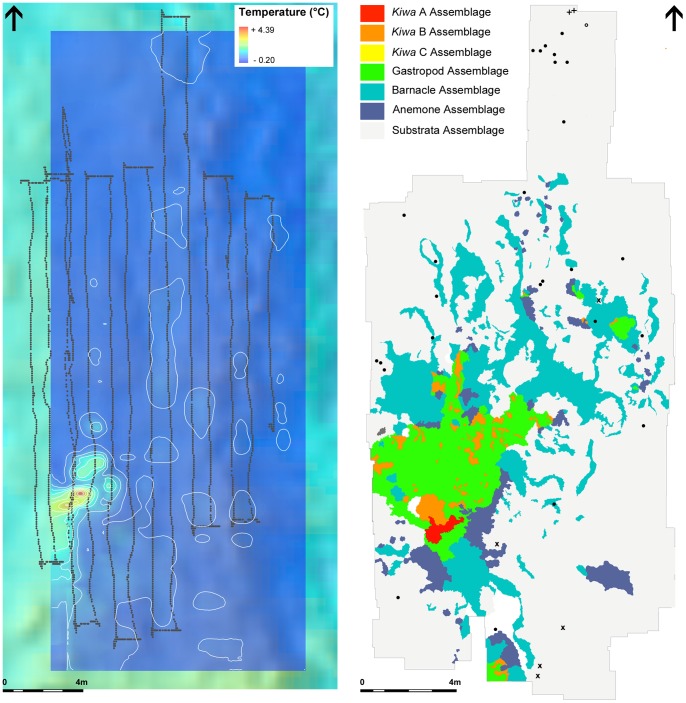
Temperature and positional data acquired from the “Twin Peaks” horizontal mosaic survey (A) Dotted line indicates the ROV position, offset to represent the 3-chip camera location. Temperature plot is an interpolation using data acquired from the ROV mounted CTD. (B) Digitised faunal assemblages of the “Twin Peaks” low-lying diffuse flow area. Assemblage types are colour coded and presented in the legend. Peripheral fauna are indicated using the following symbols (**+**) *Kiwa* n. sp. (○) unidentified Octopod (**•**) un-described Stichasterid seastar (**x**) *Colessendeis* sp. pycnogonid.

The Doppler velocity log (DVL) of the *Isis* ROV provided precise (<0.1 m in x, y, z directions) control of the position and movement of the vehicle during the acquisition of video imagery for both survey types. For surveys of horizontal surfaces, this precise control was used to maintain an altitude of 2 to 3 m above the seafloor. For surveys of vertical surfaces, the precision control of the ROV was used to maintain constant heading of camera view and maintain the position of the vehicle in a vertical plane parallel to the face of the chimney being surveyed. In both cases, DVL control was used to ensure survey lines with a minimum overlap of 50% in visible frames.

**Figure 9:**
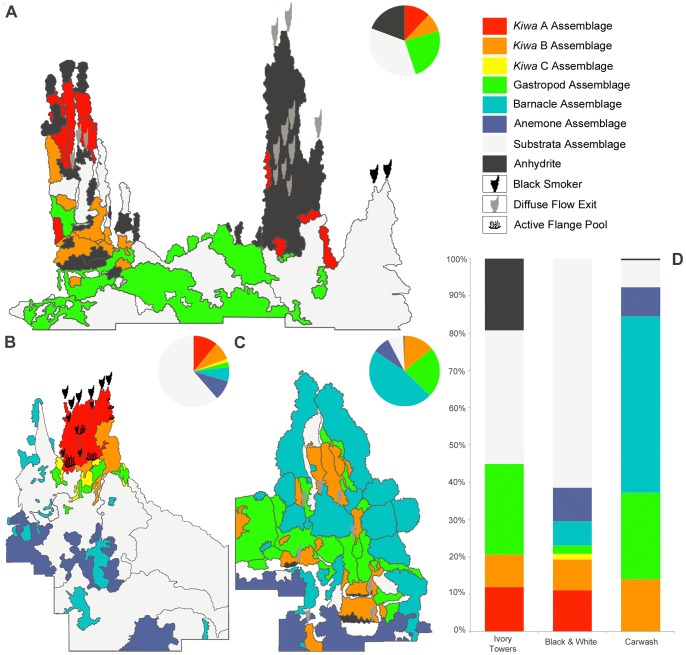
Quantitative 2D analyses for each assemblage type were performed on a comparable vertical face of each upstanding hydrothermal structure. (A) “Ivory Towers” (B) “Black & White” (C) “Carwash” (D) Comparative bar chart of percentage surface coverage of defined faunal assemblages associated with each edifice.

**Table 2:** Surface area coverage of assemblages for each mosaiced chimney structure, including minimum abundances calculations for the dominant taxa of each assemblage type based on a monolayer distribution.

	Surface Area Coverage m^2^			Minimum Abundance Dominant Fauna individuals m^−2^		
	Carwash	Black and White	Ivory Towers	Carwash	Black and White	Ivory Towers
***Kiwa*** ** A**	0.00	1.69	0.70	–	65	272
***Kiwa*** ** B**	2.46	1.27	0.51	731	533	715
***Kiwa*** ** C**	0.00	0.23	0.00	–	4017	–
**Gastropod**	4.08	0.35	1.43	1062	1781	2688
**Barnacle**	8.32	0.99	0.00	893	1686	–
**Anemone**	1.39	1.39	0.00	34	44	–
**Substrate**	1.27	9.43	2.11	–	–	–
**Anhydrite**	0.09	0.00	1.14	–	–	–
**Total Surface Area**	17.61	15.35	5.89			

Video media were then imported into a video editing software package (QuickTime Pro Version 7.6.6) and exported as a stills image sequence (full resolution 1080i HD images). These images were then used to construct mosaic images by manually aligning and superimposing frames in Adobe Photoshop CS5 extended (version 12.0×64). For mosaics of upstanding structures, where no relevant CTD data are available, video footage was reviewed simultaneously with the mosaicing process to identify and locate visible vent fluid sources including black smokers, active flanges, and areas of diffuse flow.

**Figure 10:**
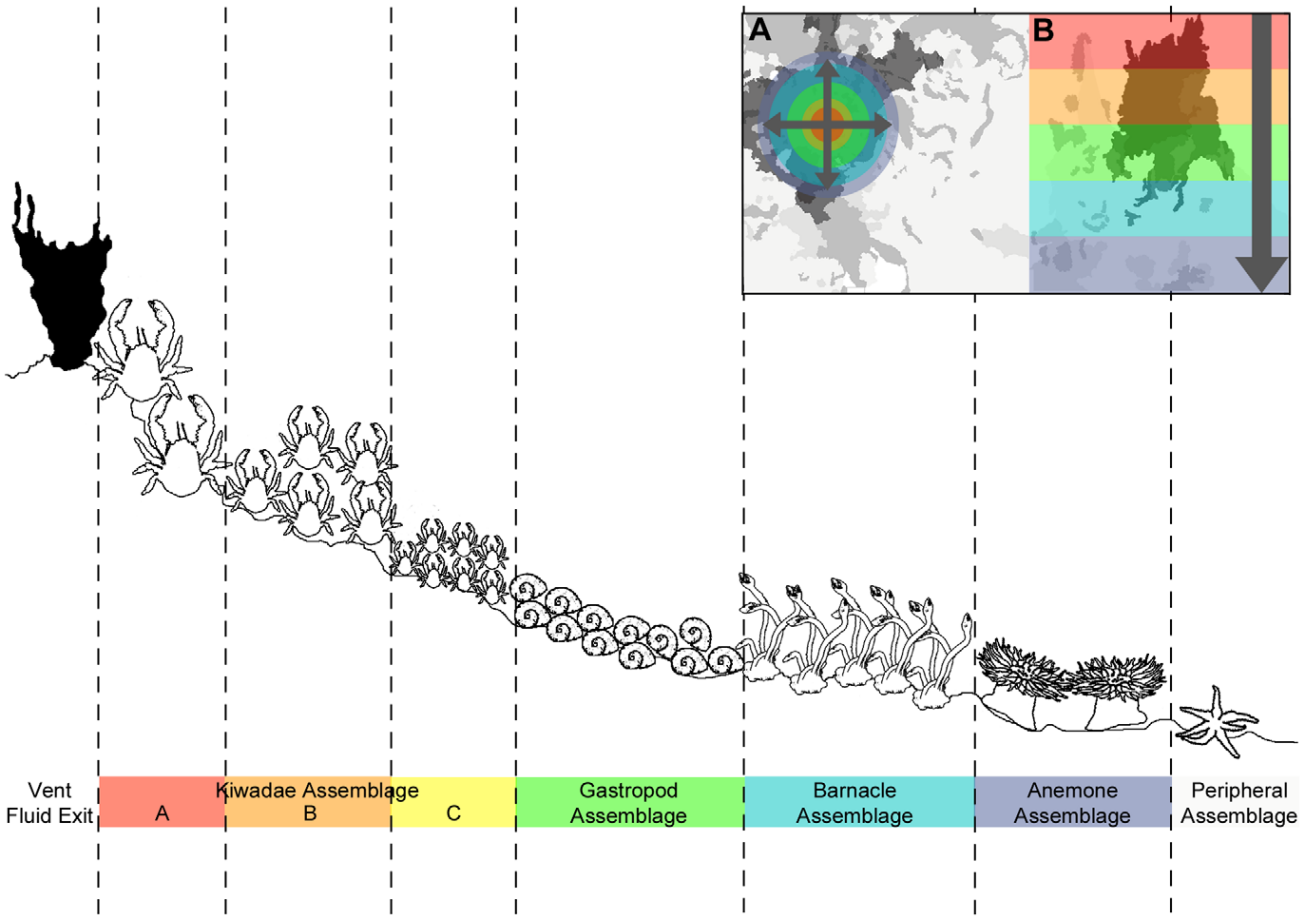
Idealised schematic of the spatial distribution of the E9 vent field faunal assemblages with increasing distance from a vent fluid exit. This spatial pattern of zonation radiates in both (A) horizontal and (B) vertical directions.

### Definition and Quantification of Faunal Assemblages

Mosaic images constructed from ROV dive footage were used to define faunal assemblage types based on dominant visible species [10], [47]. Voucher specimens of dominant species were collected during ROV dives and identified through morphological and molecular analyses [53]. From these specimens, tissue samples were dissected for stable isotope analyses.

Areas occupied by different assemblage types were defined using Photoshop to delineate their extent in mosaic images. The area of each assemblage type was determined using the 0.1 m laser scale visible in images. Percentage cover of each assemblage type was then calculated for chimney surfaces as a 2D projection of the face of the vent structure [47], using the laser scale to correct for variation in the distance between the topography of the vent chimney and the vertical plane followed by the ROV.

On the surfaces of upstanding structures, the abundances of visually-identifiable species were estimated in each assemblage type by using the 0.1 m laser scale on areas chosen to be as perpendicular to the camera view as possible. Although the mosaics were used to define and digitise the faunal assemblages on each structure, all numerical data were collated from the original stills captures extracted from the raw high-definition video footage and reviewed on a high-resolution monitor. This removes any error of double counting of mobile megafauna, which could be an artefact from the mosaicing process. For some species where multi-layered aggregations may be present, the abundances reported are a “minimum abundance” based on the visible monolayer population. Distributions of assemblage types were compared with distances from identified visible sources of vent fluids on upstanding structures.

Faunal assemblages types were also identified in the mosaic images of horizontal surfaces based on dominant visible species [50]. CTD data recorded during horizontal mosaic surveys were interpolated and contoured using Surfer 8.0 (Golden Software Inc). Interpolated data were co-registered with ROV position data in ArcMap 10 to enable comparison of distributions of assemblage types with variations in water temperature recorded by the CTD. The ROV position data were corrected to account for an offset between the locations of the CTD and camera on the vehicle.

### Stable Isotope Analyses

Approximately 0.7 mg of powder was weighed into separate tin capsules for carbon stable isotope analysis (SIA). For sulphur SIA, 2 mg of sample and 4 mg of the catalyst vanadium pentoxide were weighed into each tin capsule. Stable carbon isotope ratios were measured by continuous-flow isotope ratio mass spectrometry using a Costech Elemental Analyser interfaced with Thermo Finnigan Delta Plus XP (Natural Environment Research Council, Life Sciences Mass Spectrometry Facility, SUERC, East Kilbride, United Kingdom). Two laboratory standards were analysed every ten samples in each analytical sequence. These alternated between paired alanine standards, of differing δ^13^C, and an internal laboratory gelatin standard. Sulphur SIA was conducted by Iso-Analytical (Crewe, United Kingdom) using a SERCON Elemental Analyser coupled to a Europa Scientific 20–20 Mass Spectrometer. Laboratory standards barium sulphate (two sets of differing δ^34^S) and silver sulfide were used for calibration and drift correction. An internal standard of whale baleen was used for quality control (n = 28, 16.34‰ ± s.d. 0.21). Stable isotope ratios were expressed in delta (δ) notation as parts per thousand/permil, (‰). All internal standards are traceable to the following international standards: v-PDB (Pee Dee Belemnite), NBS-127 (barium sulphate), IAEA-S-1 (silver sulfide) and IAEA-SO-5 (barium sulphate). An external standard of freeze dried and ground white fish muscle (*Antimora rostrata*) was also analysed (δ^13^C, n = 24, −18.94‰ ± s.d. 0.09; δ^34^S, n = 30, 18.20‰, ± s.d. 0.59).

## Results

### Distribution and Setting of Vents in the E9 Vent Field

The E9 vent field (Figure 2; main) is located north of a collapse caldera (“Devil's Punchbowl”, Figure 2: top-right) on the axial high of the E9 ridge segment of the ESR, at depth ∼2400 m. The distribution of active and inactive vent chimneys (Figure 2B–2M) within the field appears to be associated with fissures parallel to the ridge axis, running NNW from the edge of the caldera across a seafloor of predominantly flat sheet lavas (Figure 2, insert A; [53]). Background seawater temperature at the E9 vent field, in areas not influenced by hydrothermal activity, is typically −1.3°C and is influenced by lower Weddell Sea Deep Water [54].

Two active chimney structures occur in close proximity at the northern limit of the vent field. “Black & White” (Figure 2D) is a ∼10 m high structure with multiple “black smoker” sources at its summit, emitting fluids with a maximum measured temperature of 380.2°C. Lower down the structure, flanges and beehives provide additional exits for hydrothermal fluids at lower temperatures. The “Black & White” structure also includes sulfide pinnacles that do not emit visible diffuse flow. A second vent chimney, “Carwash”, is located less than 5 m south of “Black & White”. “Carwash” (Figure 2E) is ∼10 m high and surmounted by two chimneys that are no longer venting high-temperature fluids from their peaks. Cooler fluids (5–19°C) and visible “white smoke” emit from sources between the two chimney structures. Towards the lower part of the chimney, flanges provide further fluid exits.

In addition to chimney structures, the northern limit of the vent field includes three areas of abundant faunal populations associated with diffuse flow from fissures in sheet lavas. Two of these areas (including “Twin Peaks” Figure 2C) lie within 100 m north of “Black & White” and “Carwash”.

The southern area of the vent field is characterised by active and extinct chimneys and diffuse flow fields distributed parallel to the ridge axis. The “Marsh Towers” structure (Figure 2H) consists of two chimneys rising from a sulfide platform, which emit diffuse flow, but no visible black-smoker venting. “Marsh Land” (Figure 2G), an area of diffuse flow from fissures in basalts lies immediately to the west. “Ivory Towers” (Figure 2I) is located ∼30 m south of “Marsh Towers” and is similarly formed of two chimney complexes on a sulfide platform. One chimney complex at “Ivory Towers” emits high temperature fluids (382.8°C) through a number of exits, including clusters of beehive diffusers. The other complex consists of five chimneys with bulbous tops that do not emit visible high-temperature fluids. Extensive venting does occur, however, from beehive diffusers and flanges at the sides of these chimneys. Several less active pinnacles also surround the main structures of “Ivory Towers”.

Two further active structures occur in close proximity ∼50 m south of “Ivory Towers”. At “Pagoda” (Figure 2J), buoyant high-temperature vent fluid pools beneath a series of flanges. Similar flange-trapped pools occur at “Launch Pad” ∼10 m to the west (Figure 2K), where there is also a single black-smoker chimney. The southernmost limit of the vent field is marked by several inactive structures, ∼100 m south of “Pagoda”: “Needle” (Figure 2L) and “Windsor Castle” (Figure 2M).

### Definition of Faunal Assemblages at E9 Vent Field

Vertical mosaic images were obtained at three vent edifices: the “Black & White” vent chimney (east and west faces; Mosaic Figure 3A), the “Carwash” vent chimney (west face; Mosaic Figure 3B), and “Ivory Towers” (north, south, east and west face; Mosaic Figure 3C). Three horizontal mosaic surveys were also completed: one at the NW corner of the “Black & White” vent chimney (resulting in a high-definition mosaic image of 17 m x 13 m of seafloor), and two in the Twin Peaks area (26 m x 7 m and 13 m x 29 m; Figure 4).

The fauna of the E9 vent field are visually dominated by four taxa: an undescribed species of anomuran crab of the genus *Kiwa*; an undescribed species of peltospiroid gastropod; an undescribed species of eolepadid barnacle of the genus *Vulcanolepas*; and undescribed species of actinostolid anemones. Initial morphological and molecular phylogenetic analyses are consistent with these taxa representing new species [53], and they are therefore referred to here as putative new species (e.g. “*Kiwa* n. sp.”). Although formal descriptions are required to confirm actual species identities, these dominant taxa can clearly be distinguished from each other in video imagery. Other fauna present among aggregations of the dominant taxa include an undescribed species of limpet of the genus *Lepetodrilus*, and at least three species of the pycnogonid genus *Sericosura*. An undescribed seven-armed sea star from the family Stichasteridae is also present towards the base of chimneys and peripheral to areas of low-temperature diffuse venting. Other common peripheral fauna include two species of the pycnogonid genus *Colessendeis*, the brisingid seastar *Freyella* sp., an unidentified species of octopus, and zoarcid fish.

Video analyses define seven assemblage types at the E9 vent field, based either on the fauna visibly dominating their biomass or the substratum type where no fauna were present (Table 1 and Figure 5; names of each faunal assemblage type are derived from their dominant taxon). In areas immediately surrounding high-temperature fluid exit, heating of seawater results in the precipitation of an anhydrite layer around the vent opening [55]. The “*anhydrite assemblage*” is typically devoid of organisms with the exception of occasional mats of white filamentous bacteria and occasional large individuals of *Kiwa* n. sp. with white filamentous bacteria growing on the carapace (Figure 5A).

Surfaces adjacent to areas of exposed anhydrite are dominated by *Kiwa* n. sp. The “*Kiwa assemblage*” can be divided into three subtypes based on average size of individuals and proximity to vent fluid exits. “*Kiwa assemblage A*” contains the largest individuals (47±0.8 mm mean carapace length.; Figure 5A), often with filamentous bacteria on their carapace when in close proximity to vent fluid sources, but individuals occur in low population densities (minimum reported 65 individuals m^−2^ for “Black & White”). At greater distance from fluid exits, but still within areas of visible diffuse flow, “*Kiwa assemblage B*” is dominated by smaller individuals (30±0.8 mm mean carapace length; Figure 5B) occurring at higher population densities (minimum 533 individuals m^−2^ for “Black & White”). Here *Kiwa* n. sp. occur in multilayer aggregations and have carapaces devoid of visible filamentous bacteria. Adjacent to “*Kiwa assemblage B*” but at greater distance from visible vent fluid sources, “*Kiwa assemblage C*” consists of juvenile specimens (12±0.4 mm mean carapace length) in abundant aggregations (minimum 4017 individuals m^−2^ for “Black & White”). In each of these assemblages, the only other visible faunal species is the limpet *Lepetodrilus* n. sp., on the carapaces of *Kiwa* n. sp. (Figure 5I). The maximum number of *Lepetodrilus* n. sp. recorded on the carapace of a collected specimen was 62 individuals and was similar to the 69 individuals counted from video imagery.

Surrounding the “*Kiwa assemblage*”, though still within areas of visible diffuse flow, the undescribed peltospiroid genus dominates the “*gastropod assemblage*” (Figure 5C), as multilayer aggregations (minimum reported abundance 1062 individuals m^−2^ for “Carwash”) and occasional chains of individuals hanging from the edges of hydrothermal structures. Other conspicuous fauna within the “*gastropod assemblage*” include *Lepetodrilus* n. sp. on the shells of the gastropods (minimum estimated abundance from video analysis was 5900–6200 individuals m^−2^; Figure 5J). Occasional individuals of *Sericosura* spp. pycnogonids and *Kiwa* n. sp. were also observed moving over the surfaces of the gastropod aggregations.

Beyond the gastropod assemblage, the fauna are visually dominated by the eolepadid cirripede *Vulcanolepas* n. sp., forming the “*barnacle assemblage*” (Figure 5D; minimum reported abundance 893 individuals m^−2^ for “Carwash”). Although the barnacles are not basally attached in areas of diffuse flow, their capitula typically occur above areas of diffuse venting. *Lepetodrilus* n. sp. occur at high abundance on the barnacles (estimates of abundances from video observations: 20 172 to 56 904 individuals m^−2^; Figure 6A), with the greatest numbers on the capitula of barnacles that are exposed to diffuse flow (Figure 6B). Actinostolid anemones and *Kiwa* n. sp. are also occasionally present among the barnacles.

Actinostolid anemones subsequently dominate the fauna at greater distance from fluid sources, defining an “*anemone assemblage*” (Figure 5E). *Pacmanactis* n. spp. are the most abundant anemones in this assemblage (minimum reported abundance 34 individuals m^−2^ for “Carwash”), but *Marianactis* n. sp. may also be present. A specimen of *Pacmanactis* n. sp. was observed egesting a carapace of *Kiwa* n. sp. (Figure 5F).

Vent structures also include some areas of visible exposed substratum but no obviously dominant or abundant faunal taxon, for example occupied by patches of visible microbial mats and sparse juvenile *Vulcanolepas* n. sp. These areas are defined as “*substratum assemblage*”. Finally, a “*peripheral assemblage*” occurs in areas where there is no visible influence of hydrothermal activity in substratum type (i.e. absence of anhydrite) or fluid sources. This assemblage includes individuals of seven-armed stichasterid seastars (Figures 5G and 5H) and fauna not present in other assemblages such as *Colossendeis* spp. pycnogonids, which were observed feeding on peripheral actinostolids (Figure 4C). Occasional small *Kiwa* n. sp. (carapace length <50 mm) were also observed in the “*peripheral assemblage*”.

### Zonation of Faunal Assemblages at E9 Vent Field

The faunal assemblages at the E9 vent field show a repeatable pattern of zonation around vent fluid sources in vertical and horizontal directions (Figure 7). The horizontal mosaic survey at “Twin Peaks” (Figures 4 and 8) show the “*Kiwa assemblage*” and “*gastropod assemblage*” associated with areas of highest temperature recorded by ROV-mounted CTD (“*Kiwa assemblage*”: up to 3.6 m maximum distance from peak temperature record; “*gastropod assemblage*”: up to 4.3 m maximum distance from peak temperature record). With increasing distance from these areas, faunal composition changes into “*barnacle assemblage*” (between 0.9 m minimum to 9.9 m maximum distance from peak temperature record) followed by “*anemone assemblage*” (between 1.1 m minimum to 13.2 m maximum distance from peak temperature record), with eventual gradation into “*peripheral assemblage*” where stichasterid seastars are prevalent (beyond 2.7 m minimum distance from peak temperature record). Stichasterids occur as solitary individuals, but also in small aggregations (closest proximity 0.11 m between centres of individual disks).

On upstanding structures where mosaic images were obtained, the same zonation is apparent with distance above, below and lateral to vent sources where substratum is available for fauna to occupy (Figure 7). In addition, the western faces of the three surveyed vent structures, (“Ivory Towers”, “Black & White” and “Carwash”) show differences in the number and type of visible vent sources (Figure 9). These structures also exhibit differences in percentage cover of assemblage types in the 2D projections of their mosaiced faces (Figure 9; Table 2).

The western face of “Ivory Towers” exhibits fifteen visible fluid flow exits across its projected mosaic area, and the highest proportion of “*anhydrite assemblage*” (Figure 9A). The “*anhydrite assemblage*” bordering high-temperature fluid sources covers 19% of the projected mosaic area. Adjacent to these patches, “*Kiwa assemblages*” account for 21% coverage (“*Kiwa assemblage A*”: 12%; “*Kiwa assemblage B*”: 9%; “*Kiwa assemblage C*”: absent; Figure 9A). The “*gastropod assemblage*” covers 24% of the total projected mosaic area, occurring towards the base of the mosaiced structure at greater distance from visible fluid flow exits. The remainder of the projected mosaic area is classified as “*substratum assemblage*” (36%). The “*barnacle assemblage*” and “*anemone assemblage*” are absent from the mosaiced area of the “Ivory Towers” structure.

 “Black & White” exhibits an archetypal chimney structure, with venting occurring from eleven visible fluid flow sources at its peak and from flanges below (Figure 9B). All assemblage types from “*Kiwa assemblage A*” to “*anemone assemblage*” are present in the mosaiced area of the western face of this structure. “*Kiwa assemblage*” types account for a total of 21% of the projected mosaic area (“*Kiwa assemblage A*”: 11%; “*Kiwa assemblage B*”: 8%; “*Kiwa assemblage C*”: 2%; Figure 9B), while the “*gastropod assemblage*” and “*barnacle assemblage*” cover 2% and 6% respectively. The “*anemone assemblage*” occupies 9%, occurring towards the base of the chimney. The remainder (62%) of the projected mosaic area at “Black & White” is represented by “*substratum assemblage*”, for example below the main active chimney where surfaces are occupied by occasional bacterial mats and sparse small barnacles.

Eight point sources of vent fluids were visible on the western side of “Carwash”, and there is no visible high-temperature venting (Figure 9C). “*Kiwa assemblage B*” covers 14% of the projected mosaic area, and occurs largely between the two relict pinnacles of the structure. Here fluid exits may be obscured from visual recognition, by dispersal of diffuse flow through dense aggregations of *Kiwa* n. sp. and neighbouring peltospiroid gastropods. The “*gastropod assemblage*” accounts for 23% of the projected mosaic area at “Carwash”. In contrast with “Ivory Towers” and “Black & White”, the “*barnacle assemblage*” accounts for 47% of the projected mosaic area at “Carwash”. Towards the base of the structure, the “*anemone assemblage*” occupies 8% of the projected mosaic area, and the “*substratum assemblage*” only represents 7%, in contrast with the other mosaiced structures. The remaining 1% of surveyed surface on this structure was represented by the “*anhydrite assemblage*”.

#### Carbon and sulphur isotope composition of dominant primary consumers

Across the faunal assemblages identified at the E9 vent field, the visually dominant taxa show an increase in δ^34^S values with distance from visible vent fluid source. *Kiwa* n. sp. sampled from the “*Kiwa assemblage*” exhibited a mean δ^34^S (s.d.) of 3.06±1.12 (n = 52), while peltospiroid gastropods from the “*gastropod assemblage*” showed a mean δ^34^S of 4.12±0.93 (n = 37), and the mean δ^34^S of *Vulcanolepas* n. sp. from the “barnacle assemblage” was 8.13±2.95 (n = 46).

The dominant primary consumers among assemblage types also varied in mean δ^13^C values. The mean δ^13^C (s.d.) value of *Kiwa* n. sp. from the “*Kiwa assemblage*” was −10.64±0.74, while the value for peltospiroid gastropods from the “*gastropod assemblage*” was −30.71±0.70. The mean δ^13^C of *Vulcanolepas* n. sp. from the “*barnacle assemblage*” was −24.46±2.52, intermediate to the values shown by the *Kiwa* n. sp. and peltospiroids.

## Discussion

### Faunal Assemblages at a Vent Field in the Southern Ocean

The fauna occupying the E9 hydrothermal vent field on the East Scotia Ridge belong to a proposed new Southern Ocean province of vent biogeography [53]. The assemblages at the E9 vent field include aggregations of a new species of anomuran crab, *Kiwa* n. sp., and video mosaicing of vent structures shows that this species occurs at abundances of >700 individuals m^−2^ in close proximity to vent fluid sources. Two other species of *Kiwa* are known to occur in chemosynthetic environments. *Kiwa hirsuta* occurs at hydrothermal vents on the Pacific-Antarctic Ridge, but at lower population densities (0.1 to 0.2 individuals m^−2^) towards the periphery of vent fields [56]. *Kiwa puravida* occurs at cold seeps on the Costa Rica margin, but has not been observed in extensive aggregations similar to those of the *Kiwa* species at the E9 vent field [57]. Recently, specimens superficially resembling *Kiwa* n. sp. have also been found in close proximity to active vent sources at a vent field on the SW Indian Ridge, though at population densities at least an order of magnitude lower than those observed at the E9 vent field in the Southern Ocean (Copley & Marsh, pers. obs.). The galatheid *Shinkaia crosnieri* found in the Okinawa Trough back-arc basin of the western Pacific [58] is the only other anomuran known to occur in dense aggregations (exceeding 560 individuals m^−2^) in close proximity to vent fluid sources [51].

The ventral surface of *Shinkaia crosnieri* is covered in plumose setae, similar to *Kiwa* n. sp., and *S. crosnieri* has been observed “combing out” these setae using its third maxilliped to transfer epibiotic bacteria to its mouth [68]. Carbon radioisotope uptake and stable isotope studies confirm that *S. crosneiri* obtains nutrition from harvesting epibiotic bacteria in this fashion [69], [70]. *Kiwa hirsuta* and *K. puravida* are thought to harvest epibiotic bacteria similarly from the setae on their chelipeds, although this has only been demonstrated for *K. puravida* [57]. The chelipeds of *Kiwa* n. sp. are not conspicuously setose and much shorter in proportional length than those of *K. hirsuta* and *K. puravida*, but its ventral surface is densely covered in setae unlike other *Kiwa* species. Filamentous bacteria associated with these ventral setae suggest that *Kiwa* n. sp. may also feed on epibiotic bacteria [53].

Assemblages dominated by other decapod crustaceans occur in close proximity to vent fluid sources at some Atlantic and Indian Ocean vents. Alvinocarid shrimp of the genus *Rimicaris* occur at high densities close to vent sources at depths greater than 3000 m on the Mid-Atlantic Ridge (*R. exoculata*, 1500–2500 individuals m^−2^; [45], [59], [60]), on the Central Indian Ridge (*R. kairei*; [61], [62], [63], [64]), and on the Mid-Cayman Spreading Centre (*R. hybisae*, 2000 individuals m^−2^; [65], [66]). In contrast, the substratum adjacent to high-temperature fluid exits on vent chimneys at east and northeast Pacific ridge vent fields is typically occupied by alvinellid polychaetes (∼2000 individuals m^−2^; [67]).

Among taxa occupying a similar position in faunal zonation at vents in other biogeographic provinces, *Rimicaris exoculata* are also thought to derive nutrition from epibiotic bacteria [60], [71], [72], which may include methanotrophs at vent fields in ultramafic settings [73], and a nutritional role of epibiotic bacteria is also indicated for the polychaete *Alvinella pompejana* [74]. The δ^13^C values of *Kiwa* n. sp. sampled from the “*Kiwa assemblage*” at the E9 vent field are similar to values found in other vent species thought to feed on an epibiont flora dominated by epsilon-Proteobacteria [59], [70], [74], [75], although the epibiont flora associated with the ventral setae of *Kiwa* n. sp. have not yet been characterised.

The occurrence of *Kiwa* n. sp. at the E9 vent field extends from aggregrations around vent fluid sources to individuals in peripheral areas. Direct temperature probe measurements of the “*Kiwa assemblages*” defined by video mosaicing of vent structures ranged from 10.1 to 12.6°C. Lower temperatures of −0.11 to 1.02°C were recorded by the ROV-mounted CTD where individual *Kiwa* n. sp. occur in the periphery of the vent field (Figure 8), but these temperature data are not directly representative of conditions inhabited by the animals because of the elevated position of the CTD sensor on the ROV. Lithodid crabs are the other anomuran taxon known to maintain adult populations in deep Antarctic waters south of the Polar Front [76], although a squat lobster, *Munidopsis albatrossae,* has also been recorded from the Bellingshausen Sea [77]. However, lithodid crabs appear to be excluded at temperatures lower than 0.5°C [78]. Explanations for a general absence of reptant decapods from deep Antarctic waters include their inability to regulate haemolymph magnesium concentrations, resulting in loss of activity and death at cold temperatures [79], [80]. The elevated temperatures recorded directly by temperature probe in areas occupied by “*Kiwa assemblages*” may exclude these aggregations from this limitation.

The “*gastropod assemblage*” at the E9 vent field is visually dominated by an undescribed species of peltospiroid and occurs adjacent to “*Kiwa assemblages*” but, at greater distance from visible vent fluid sources. Peltospiroid gastropods are a geographically widespread taxon at hydrothermal vents, and occur in a similar position in vent faunal zonation at several vent fields on the Central Indian Ridge (undescribed “scaly foot” gastropod; [62], [64]) and on the Juan de Fuca Ridge in the northeast Pacific (*Depressigyra globulus*; [10], [81]). At the 9°N vent field on the East Pacific Rise, three species of peltospiroid are associated with the “alvinellid zone” but do not dominate its assemblages [36]. Two morphospecies of peltospiroid are also abundant at newly-explored vents on the SW Indian Ridge (Copley & Marsh, pers. obs.), and another peltospiroid occurs in high abundance at a vent field north of the Azores on the Mid-Atlantic Ridge [82]. Physico-chemical tolerances are thought to be important in defining gastropod microdistributions within hydrothermal vent fields, although other factors such as biological interactions and microbial flora may also be important [36], [38], [81]. The δ^13^C values of peltospiroid gastropods from the “*gastropod assemblage*” are similar to those of other taxa with endosymbionts dominated by gamma-Proteobacteria, such as the gastropod *Ifremeria nautilei* [83] and some *Bathymodiolus* spp mussels [75], [84], [85].

The sessile filter-feeding stalked barnacle *Vulcanolepas* n. sp. dominates the third assemblage away from visible vent fluid sources in video mosaics at the E9 vent field. Eolepadid barnacles occupy a similar position in faunal zonation at vent fields on the Central Indian Ridge [62], [64], [75], SW Indian Ridge (Copley & Marsh, pers. obs), and several back-arc basins of the western Pacific [86], [87] where similar abundances have been recorded to those reported at the ESR [88].

Molecular phylogenetics of *Vulcanolepa*s n. sp. from the E9 vent field indicates that it is most closely related to *V. osheai* from the Brothers Caldera on Kermadec Ridge in the SW Pacific [53]. The cirral setae of *V. osheai* harbour filamentous bacteria, and stable isotope and fatty acid analyses indicate that *V. osheai* derives nutrition from these epibionts [87]. Eolepadid barnacles at other vents also harbour filamentous bacteria on elongated cirral setae and may be capable of feeding on finer particles than other deep-sea barnacles [86]. The stable isotope composition of eolepadid barnacles from vents on the Central Indian Ridge is consistent with nutrition from epibiotic bacteria in addition to filter feeding [75]. The δ^13^C values of *Vulcanolepas* n. sp. from the “barnacle assemblage” suggests a similar mixed nutritional mode for eolepadid barnacles at the E9 vent field.

In the “*barnacle assemblage*” and “*gastropod assemblage*” defined by video mosaicing, lepetodrilid limpets are the numerically dominant species, occurring at high abundances on the peduncles and capitula of the eolepadids, the shells of the peltospiroids, and also occasionally on the carapaces of individual *Kiwa* n. sp., both in the “*Kiwa assemblages*” and in the periphery of the E9 vent field. Lepetodrilids are a numerically dominant component of the fauna in zones comparable to the “*barnacle assemblage*” at some vent fields in other regions, for example on the northern East Pacific Rise [11] and Juan de Fuca Ridge [81]. Although the proximity of lepetodrilid species closest to vent sources may be determined by tolerance of hydrothermal conditions [89], their distribution is often widespread across other faunal assemblages at vent fields elsewhere [36].

The “*anemone assemblage*” defined by video mosaicing occurs beyond the “*barnacle assemblage*” at greater distance from visible vent fluid sources in the E9 vent field, and is the most peripheral assemblage type dominated by a single taxon. Actinostolids occupy a similar position in faunal zonation at several vent fields in other biogeographic provinces, either dominating peripheral zones (e.g. TAG hydrothermal mound, Mid-Atlantic Ridge [90]; Rose Garden, Galapagos Rift [9]; Kairei and Edmond fields, Central Indian Ridge [61], [62]) or surfaces at low temperatures (Ashadze-1, Mid-Atlantic Ridge [49]). The abundances of actinostolids in the “*anemone assemblage*” at the E9 vent field (33–44 individuals m^−2^) are comparable with anemone abundances reported on the Mid-Atlantic Ridge at the TAG hydrothermal mound (∼20 individuals m^−2^; [91]) and Ashadze-1 (32 individuals m^−2^; [49]), and at the Beebe Vent Field on the Mid-Cayman Spreading Centre (>20 individuals m^−2^; [66]). The observation of an anemone egesting a carapace of *Kiwa* n. sp. at the E9 vent field is consistent with a general recognition of actinostolids as secondary consumers at vents [92].

The “*peripheral assemblage*” defined by horizontal video mosaicing at the E9 vent field is characterised by the occurrence of secondary consumers such as octopus, seven-armed stichasterid asteroids and *Colessendeis* spp. pycnogonids (Figure 4A, 4B and 4C). Specimens of the suspension-feeding brisingid genus *Freyella* were also present on inactive structures in the southern sector of the E9 vent field (“Needle” and “Windsor Castle”, Figures 2L and 2M). Predators, scavengers and suspension-feeders known from non-vent environments are known to occur opportunistically in the periphery of vent fields [93]. Whether taxa only observed in the “*peripheral assemblage*” at the E9 vent field can be considered “vent endemic” is therefore not clear, even where stable isotope values indicate nutrition ultimately derived from chemosynthetic sources of carbon fixation [94].

Overall, although the fauna present at the E9 vent field are distinct from those of other biogeographic provinces at species level, it is clear that they exhibit similarities in zonation patterns at higher taxonomic levels. Similarities in zonation at disparate locations may result from family-level constraints of physical tolerances to physico-chemical gradients at vents, and/or family-level similarities in trophic ecology. However, the families that dominate the assemblages at the E9 vent field do not always co-occur at vents in other biogeographic provinces. Currently, a zonation dominated by peltospiroids, eolepadids, and actinostolids with increasing distance from vent sources is only known at vent fields on the Central Indian Ridge [62], [64]. At family level, the E9 vent field is distinct in the high abundance of kiwaid crabs in close proximity to high-temperature vent sources, not previously observed for other kiwaid species, and only known for one other anomuran species at vent examined so far [51].

### Microdistribution Patterns Revealed by Large-scale Video Mosaicing at a Vent Field

There is a consistent pattern of faunal zonation at the E9 vent field, transitioning between assemblage types with increasing distance from vent fluid sources (Figure 10). Faunal zonation occurs in both vertical and horizontal directions around “black smoker” sources and areas of visible diffuse flow (Figure 7). The importance of thiothrophic nutrition for primary consumers may decline with distance from vent fluid sources, as indicated by the increase in δ^34^S values across the zonation of their assemblages [95]. There are variations, however, in the coverage of surfaces by different assemblage types on individual chimneys surveyed at the E9 vent field (Figure 9). These chimneys vary in the occurrence of visible fluid sources and predominance of focused high-temperature venting or lower-temperature diffuse flow.

The western face of “Ivory Towers” exhibits the highest concentration of high-temperature fluid flow exits and the highest proportion of “*anhydrite assemblage*” among the structures surveyed, and the faunal zonation only extends to the “*gastropod assemblage*” in the mosaiced area. At “Black and White”, most venting occurs as “black smoker” activity at the peak of the structure, immediately surrounded by “*Kiwa assemblages*”. With most sources of buoyant vent fluid concentrated at the top of this structure, much of the lower chimney is uncolonised by faunal assemblages, with small patches of “*barnacle assemblage*” on relict pinnacles above other vent fluid sources.

At “Carwash”, there are no visible sources of high-temperature vent fluids, and no visible venting at the peaks of its two sulfide pinnacles. Instead, venting occurs as diffuse flow from the central and lower portions of the structure. More than 90% of its surveyed area is covered by faunal assemblages, dominated by the “barnacle assemblage” (Figure 9D). Zonation from “*Kiwa assemblage*” to “*barnacle assemblage*” occurs repeatedly across the lower structure around individual sources of visible vent fluids. Dense faunal aggregations also disperse vent fluids laterally from their sources in this area, and there may be additional fluid flow exits in this area that are obscured by faunal aggregations. A similar dispersion of vent fluids by mussel beds at Rose Garden on the Galapagos Rift has been proposed to extend the seawater-effluent interface and thereby increase habitat for vent fauna [15].

Surveying several structures with different level and types of activity in the E9 vent field reveals a possible successional pattern in faunal colonisation as activity at a particular structure declines. Nascent vent structures with high levels of “black smoker” venting may resemble “Ivory Towers” in assemblage types and coverage, while structures where high-temperature activity is waning may resemble “Carwash”. “Black & White” may represent a transition between these two extremes. This successional pattern can be tested on future visits to the E9 vent field, using the large-scale high-definition video mosaics compiled here as a baseline to assess faunal change.

Variations in faunal microdistribution patterns at the E9 vent field appear to be aligned with temperature gradients, where CTD temperature data are available for large-scale mosaic surveys of horizontal surfaces (Figure 8). Temperature is often used as a proxy for physico-chemical conditions at vents, and considered to represent the primary abiotic drivers in the spatial structuring of vent assemblages (e.g., [20], [50], [81], [96], [97], [98]). However, biological processes may also be important in structuring faunal assemblages in vent environments (e.g., [8], [23], [44], [71], [99], [100], [101], [102]), but subsequent investigation of these processes usually requires ecological experimentation at the seafloor [7].

Here, large-scale, high-definition video mosaicing at the E9 vent field has enabled detailed determination and quantification of faunal zonation and microdistribution patterns over a similar spatial scale to studies in accessible intertidal environments. Determining patterns of spatial variation is a prerequisite for elucidating processes [6] and a necessary first step in understanding the ecology of a new province of vent biogeography. Although the presence/absence of species is used to distinguish biogeographic provinces, such information does not represent all aspects of ecological similarity or difference. There are some similarities at family level in the zonation of assemblages at the E9 vent field and those of other provinces, but also some differences. Determining such patterns at a global scale, and understanding their genesis, represents a goal for vent ecology beyond the mapping of biogeographic distributions.
